# Evaluating the efficacy and safety of different neoadjuvant immunotherapy combinations in locally advanced HNSCC: a systematic review and meta-analysis

**DOI:** 10.3389/fimmu.2024.1467306

**Published:** 2024-08-29

**Authors:** Chang Liu, Mingzhu Li, Xiaojie Liu, Ting Shi, Yun Wang, Chaoyang Sui, Wenan Zhang, Bowen Wang

**Affiliations:** ^1^ Department of Burns and Plastic Surgery, Yantaishan Hospital, Yantai, China; ^2^ Department of implantology, Affiliated Hospital of Binzhou Medical College, Yantai Stomatology Hospital, Yantai, China

**Keywords:** HNSCC, neoadjuvant immunotherapy, efficacy, safety, meta-analysis

## Abstract

**Background:**

Immune checkpoint inhibitors have demonstrated promising therapeutic outcomes in recurrent/metastatic (R/M) Head and Neck Squamous Cell Carcinoma (HNSCC), prompting numerous clinical trials to investigate the safety and efficacy of this approach in neoadjuvant therapy. This systematic review aims to consolidate and analyze the findings from various clinical trials combining neoadjuvant immunotherapy for HNSCC, with the goal of identifying the most effective neoadjuvant immunotherapy regimen.

**Methods:**

The system conducted searches across electronic databases including PubMed, Embase, the Cochrane Library and Web of science from their inception to July 1, 2024. The primary focus was on evaluating efficacy (particularly pathological complete response (pCR), major pathological response (MPR), and overall response rate (ORR)) and safety (primarily assessed by grade 3-4 treatment-related adverse reactions).

**Results:**

A total of 1943 patients from 32 studies were analyzed. Combining neoadjuvant immunotherapy with chemotherapy or radiotherapy demonstrated superiority over neoadjuvant immunotherapy alone in terms of the MPR rate, while showing no statistically significant difference in the pCR rate. Furthermore, the combination of neoadjuvant immunotherapy with chemotherapy or radiotherapy exhibited a lower CR rate compared to neoadjuvant immunotherapy with radiotherapy alone, but a higher PR rate and SD rate. Apart from the neoadjuvant immunotherapy group in isolation, there were no statistically significant differences in grade ≥3 treatment-related adverse events (TRAEs) and immune-related adverse events (irAEs) among the other three combination therapy groups.

**Conclusion:**

This systematic review and meta-analysis indicate that patients with locally advanced HNSCC might benefit from neoadjuvant immunotherapy, particularly when used in conjunction with chemotherapy or radiotherapy. Nonetheless, additional data is required to definitively confirm its efficacy.

**Systematic Review Registration:**

https://www.crd.york.ac.uk/PROSPERO/display_record.php?RecordID=553753, identifier CRD42024553753.

## Introduction

Head and neck squamous cell carcinoma (HNSCC) arises in the mucosal epithelium of the oral cavity, pharynx, and larynx, representing the most prevalent form of cancer within the head and neck. This region is anatomically intricate, serving crucial roles in essential functions such as eating, speaking, and breathing (1). The majority of HNSCC patients receive a diagnosis of localized or locally advanced disease, with standard treatment typically involving a combination of radiotherapy, surgery, and possibly chemotherapy tailored to individual risk levels (2). However, individuals diagnosed with locally advanced HNSCC face a significant risk of both local recurrence (approximately 15-40%) and distant metastasis, with a 5-year overall survival rate of only 50% (3). While platinum-based chemotherapy, like the Docetaxel + cisplatin + 5-fluorouracil (5-FU) regimen, is the standard neoadjuvant treatment for HNSCC patients, research indicates that these strategies may not always effectively extend patient survival or prevent progression due to insensitivity or resistance to these chemotherapeutic agents (4–6). Novel treatment approaches are essential to enhance survival rates or lessen the burden of conventional therapies.

Recently, the academic community has increasingly acknowledged the efficacy of immune checkpoint inhibitors, specifically monoclonal antibodies targeting programmed cell death-1 (PD-1) and programmed cell death ligand-1 (PD-L1), in managing relapsed or metastatic HNSCCs. Preclinical studies indicate that neoadjuvant PD-1/PD-L1 pathway blockade may be more effective than adjuvant blockade, leveraging tumor antigens within the preoperative immune environment for enhanced efficacy (7, 8). In a phase Ib study, the effectiveness and safety of neoadjuvant immunoradiotherapy in patients with locally advanced HNSCC were highlighted, demonstrating an MPR of 86%, a complete pathologic response of 67%, and a clinical-to-pathologic downstaging rate of 90% (9). Several current trials investigating neoadjuvant immunotherapy for HNSCC, focusing on single or dual immunotherapy, as well as combinations with chemotherapy or radiotherapy, have displayed encouraging outcomes (10–12).

This meta-analysis endeavors to gather findings from current clinical studies to evaluate the effectiveness and safety of various neoadjuvant immunotherapy combination treatments for managing locally advanced HNSCC, offering additional clinical treatment alternatives.

## Methods

This systematic review adheres to the Preferred Reporting Items for Systematic Reviews and Meta-Analyses (PRISMA) guidelines (13). The comprehensive protocol has been registered online with the International Prospective Register of Systematic Reviews (PROSPERO: CRD42024553753). As this review and meta-analysis did not involve the use of individual patient data, it was not subject to institutional review board approval.

### Search strategy and study selection

We systematically searched databases including PubMed, Embase, the Cochrane Library and Web of science for relevant studies published before July 2024 concerning neoadjuvant immunotherapy in patients with HNSCC (refer to **Supplementary Materials** for the search strategy). Additionally, we sought unpublished data from ongoing clinical trials on neoadjuvant immunotherapy in HNSCC patients presented at major international oncology conferences such as the American Society of Clinical Oncology and the European Society of Oncology Medicine.

### Selection criteria and data extraction

This analysis included clinical trials investigating immunotherapy as a neoadjuvant intervention in HNSCC patients without distant metastases. Patients with potentially curable primary lesions in the oral cavity, oropharynx, hypopharynx, and larynx (excluding the nasopharynx) were considered. Two researchers (CL and MZL) independently screened and extracted articles for potential inclusion. In cases of disagreement, a discussion or consultation with a third researcher was conducted to determine study inclusion. Data were meticulously documented and stored in an Excel spreadsheet. Parameters were extracted in a standardized format, including details such as the first author, publication year, approval number, study design (single-arm or randomized controlled trial), pathological stage, treatment regimen, sample size, age distribution, gender ratio, pathological complete response (pCR), major pathological response (MPR), R0 resection rate, incidence of grade 3 or higher treatment-related adverse events (TRAEs), complete response (CR), partial response (PR), overall response rate (ORR), stable disease (SD), disease control rate (DCR), and other relevant factors.

### Statistical analysis

The meta-analysis was conducted utilizing non-comparative binary data from RevMan software version 5.4 (Cochrane Collaboration), given that the majority of studies were single-arm clinical trials. Effect indicators such as odds ratios (ORs) and their corresponding 95% confidence intervals (CIs) were employed. Subgroup analysis was carried out based on different combination treatment approaches. Statistical heterogeneity was assessed using the Cochran Q chi-square test and the inconsistency index. In cases where study heterogeneity was low (P > 0.1, I^2^ < 50%), a fixed-effect model was applied. Conversely, if significant heterogeneity was present, the random-effects model was utilized.

### Study quality

The two reviewers utilized the MINORS scale to evaluate the study quality. This scale is specifically tailored for assessing non-randomized studies and comprises 8 criteria, each rated on a scale of 0-2, resulting in a total score of 16. Studies scoring between 13-16 points were classified as high-quality, those scoring 9-12 points were deemed moderate quality (and included in the final analysis and data extraction), while studies scoring below 9 points were regarded as low quality and therefore excluded from the analysis.

## Results

### Characteristics of included studies

**Figure 1:**
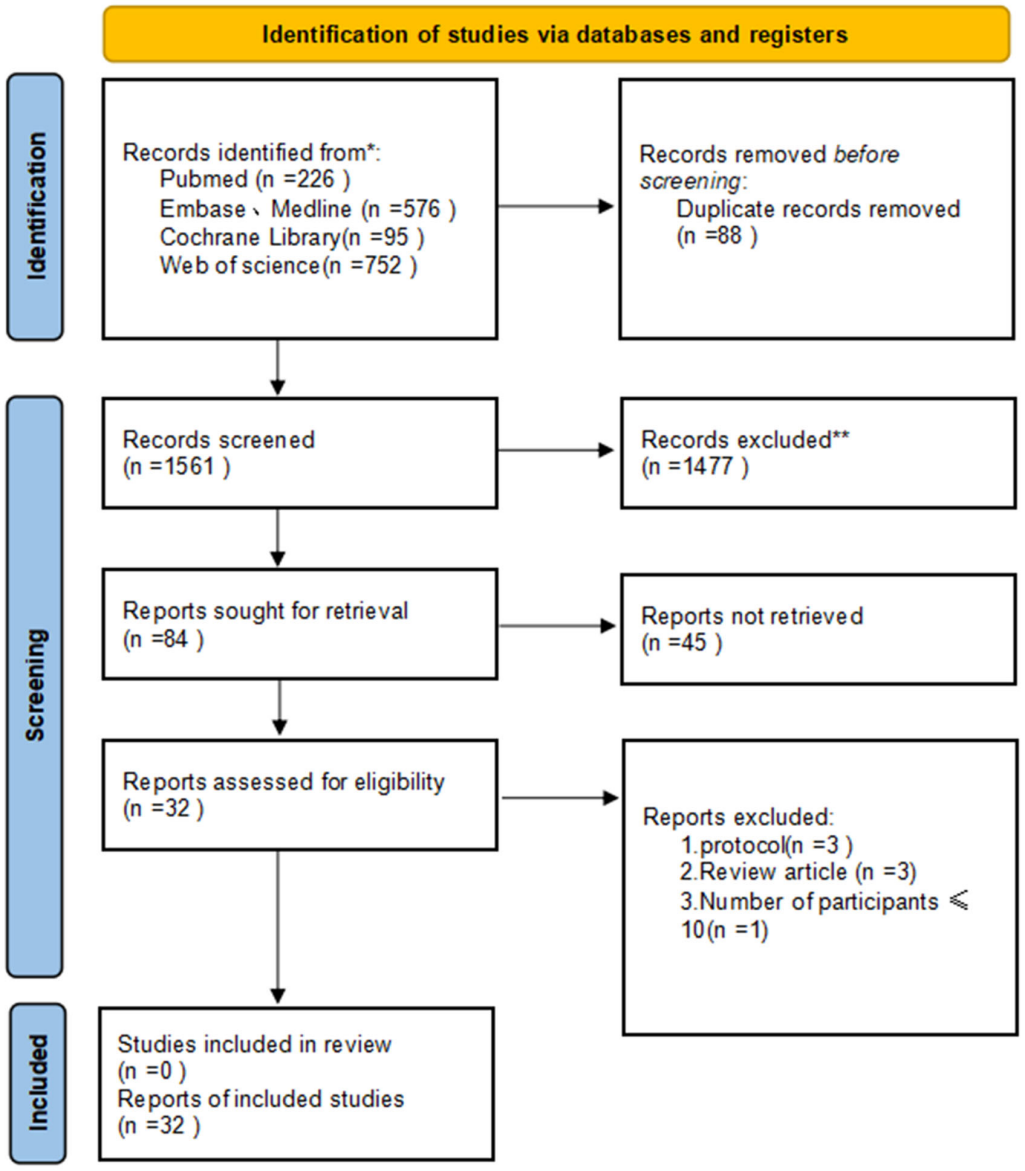
Preferred Reporting Items for Systematic Reviews and Meta-Analyses (PRISMA) diagram of the study selection.

The PRISMA diagram illustrating the selection process is detailed in **Figure 1**. Following the search strategy, a total of 1649 studies were screened, with 88 duplicates removed. Among the 32 selected studies, encompassing 1943 patients, all met the criteria for inclusion in the final meta-analysis. Notably, four of these studies were in the form of conference abstracts. The meta-analysis comprised 23 single-arm clinical studies and 9 randomized controlled trials, categorized based on different combination therapy modalities: 11 (10, 14–23) studies focused on neoadjuvant immunotherapy alone (NI), 12 (1, 11, 24–33) studies on neoadjuvant immunotherapy combined with chemotherapy (NICT), 5 (9, 34–37) studies on neoadjuvant immunotherapy combined with radiotherapy (NIRT), and 4 (12, 38–40) studies on neoadjuvant immunotherapy combined with chemoradiotherapy (NICRT). **Table 1** summarizes the key characteristics of the included studies, while the main outcomes are presented in **Supplementary Table 1**. Additionally, **Supplementary Table 2** indicates an overall low risk of bias across the included studies.

**Table 1:** Study features of neoadjuvant immunotherapy for head and neck squamous cell carcinoma.

Author year	NCT number	Study design	TYPE	Article type	Combination therapy	Clinical stage	No. of patients
Renata Ferrarotto2020 (14)	NCT03144778	NI	RCT	Full text	Durvalumab+Tremelimumab	II-IVA	29
Ravindra Uppaluri2020 (15)	NCT02296684	NI	Single-arm	Full text	Pembrolizumab	III-IVB	36
L. Zuur 2020 (16)	NCT03003637	NI	Single-arm	Full text	Nivolumab+/Ipilimumab	II-IVB	32
Renata Ferrarotto2021 (17)	NCT03565783	NI	Single-arm	Full text	Cemiplimab	III-IVA	20
Robert L Ferris2021 (18)	NCT02488759	NI	Single-arm	Full text	Nivolumab	III–IV	52
Hannah M. Knochelmann2021 (19)	NCT03021993	NI	Single-arm	Full text	Nivolumab	II-IVA	12
Joris L. Vos2021 (20)	NCT03003637	NI	Single-arm	Full text	Nivolumab+Ipilimumab	II-IVB	32
Glenn J. Hanna2022 (21)	NCT03341936	NI	Single-arm	Full text	Nivolumab+Lirilumab	I-IVb	28
Wu-tong Ju2022 (22)	NCT04393506	NI	Single-arm	Full text	Camrelizumab+Apatinib	III-IVB	21
Trisha M. Wise-Draper2022 (23)	NCT02641093	NI	Single-arm	Full text	Pembrolizumab	III- IV	92
Chang Gon Kim2022 (10)	NCT03737968	NI	RCT	Conference abstract	Durvalumab+/Tremelimumab	Locally advanced stage	45
R. Zinner2020	NCT03342911	NICT	Single-arm	Full text	Nivolumab+Carboplatin + paclitaxel	III-IV	26
Markus Hecht2020 (25)	NCT03426657	NICT	Single-arm	Full text	Tremelimumab+Cisplatin (carboplatin)/Docetaxel	III-IVB	56
Konstantin Hellwig 2021 (26)	NCT03426657	NICT	Single-arm	Full text	Tremelimumab/durvalumab+Cisplatin/Docetaxel	III-IVB	22
Xia Li2021	No.201356HN	NICT	RCT	Full text	Sintilimab + docetaxel + platinum + fluorouracil	cT1-2 N1-3/cT3-4 N0-3	65
Markus Hecht2022 (28)	NCT03426657	NICT	Single-arm	Full text	Durvalumab+tremelimumab+Cisplatin + Docetaxel	III–IVB	79
Xiaotao Huang2022 (29)	NCT04947241	NICT	Single-arm	Full text	Toripalimab+ gemcitabine + cisplatin	III–IVB	23
Zhanjie Zhang2022 (30)	ChiCTR1900025303	NICT	Single-arm	Full text	Camrelizumab+ albumin with paclitaxel/docetaxel + cisplatin	III–IVB	30
Kai Wang2023 (31)	ChiCTR2200055719	NICT	Single-arm	Full text	Pembrolizumab+Cisplatin + paclitaxel	III-IV	22
Di Wu2024 (1)	NCT04826679	NICT	Single-arm	Full text	Camrelizumab+ paclitaxel + cisplatin	II-IV	48
Ralph Zinner2020	NCT03342911	NICT	Single-arm	Conference abstract	Nivolumab + carboplatin + paclitaxel	III-IV	27
Wang, H2023 (32)	NCT05522985	NICT	RCT	Conference abstract	Topalizumab + paclitaxel + cisplatin	III-IV	52
Wang Hongling2024 (33)	NCT 05522985	NICT	RCT	Full text	Triplimab + albumin paclitaxel + cisplatin	III-IV	23
Rom Leidner2021 (9)	NCT03247712	NIRT	Single-arm	Full text	Nivolumab+Stereotactic whole body Radiation Therapy (SBRT)	Locally advanced stage	21
Laurel B. Darragh2022 (34)	NCT03635164	NIRT	Single-arm	Full text	Durvalumab+SBRT	II-IV	21
Peng Shen2022 (35)		NIRT	Single-arm	Full text	Nivolumab+SBRT	III-IVB	30
Jennifer M Johnson2023 (36)	NCT03162731	NIRT	Single-arm	Full text	Nivolumab+ipilimumab+ radiotherapy	IVA-IVB	24
Mell, L. K.2022 (37)	NCT03258554	NIRT	RCT	Conference abstract	Durvalumab+ radiotherapy	III-IV	123
Steven F. Powell2020 (38)	NCT02586207	NICRT	Single-arm	Full text	Pembrolizumab+ cisplatin + radiotherapy	III-IVB	59
Yungan Tao2020 (39)	NCT02999087	NICRT	RCT	Full text	Avelumabe + cetuximabe + radiotherapy	III-IV	41
Nancy Y Lee2021 (12)	NCT02952586	NICRT	RCT	Full text	Avelumab+ chemoradiotherapy	IVA-IVB	350
Jean-Pascal Machiels2024 (40)	NCT03040999	NICRT	RCT	Full text	Pembrolizumab + chemoradiotherapy	IVA-IVB	402

NI, Neoadjuvant immunotherapy; NICT, Neoadjuvant immunotherapy combined with chemotherapy; NIRT, Neoadjuvant immunotherapy combined with radiotherapy; NICRT, Neoadjuvant immunotherapy combined with chemotherapy and radiotherapy.

### Evaluation of efficacy outcomes

#### Pathological response

**Figure 2:**
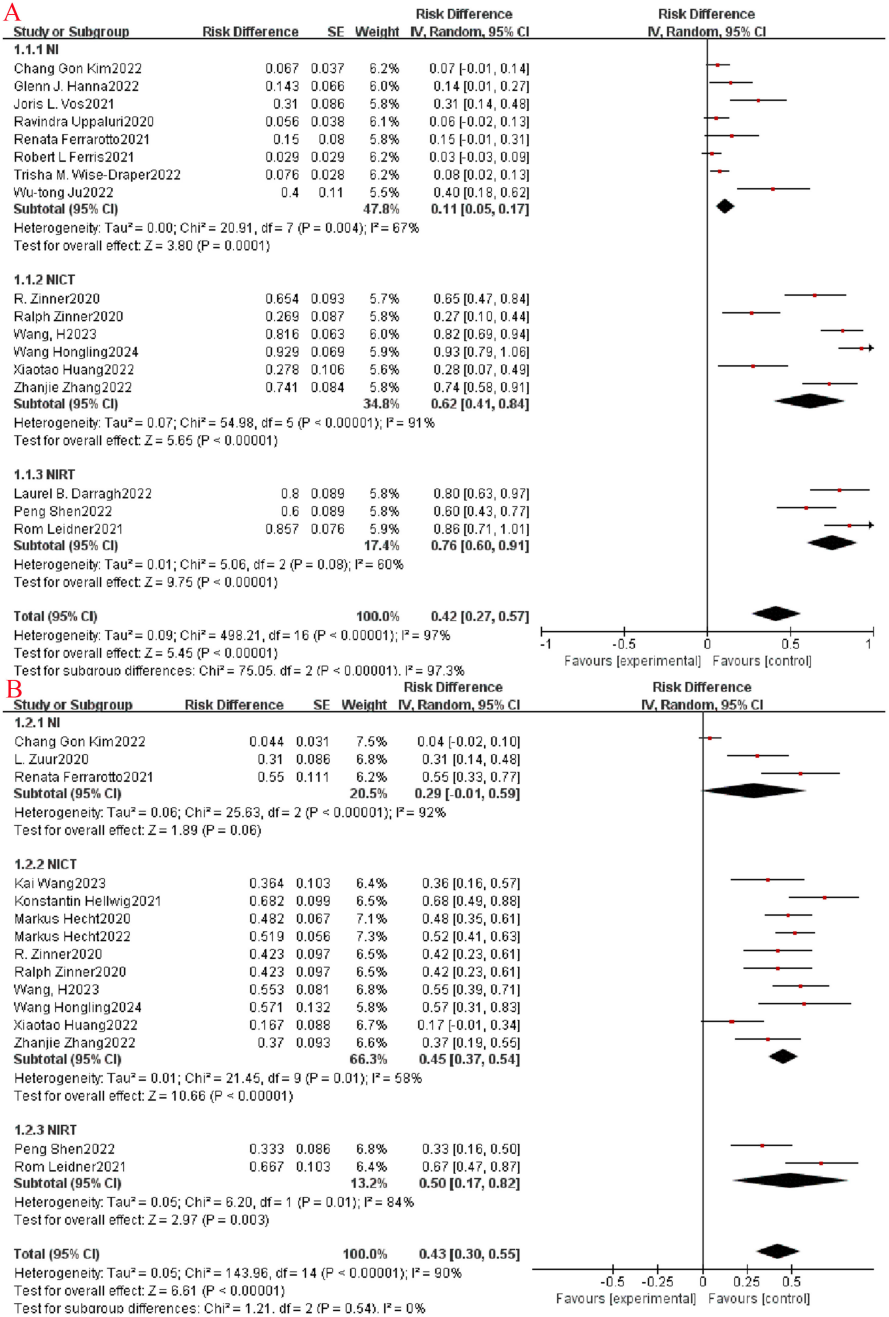
Neoadjuvant immunotherapy efficacy forest plot. **(A)**:MPR;**(B)**:pCR.

This study primarily assessed the efficacy of neoadjuvant immunotherapy by analyzing MPR and pCR rates. Across the enrolled studies, MPR rates varied widely from 2.9% to 92.9%. Among the 17 qualifying studies, subgroup analysis revealed a notably higher MPR rate in the NIRT group (OR=0.76, 95% CI: 0.60-0.91, P< 0.0001, I^2^ = 97.3%, **Figure 2A**) compared to the NI and NICT groups. Furthermore, the 15 studies that reported pCR rates (ranging from 16.7% to 68.2%) indicated that both the NIRT and NICT groups had higher pCR rates than the NI group, although this difference did not reach statistical significance (P=0.54, I^2^ = 0%, **Figure 2B**).

#### Radiological response

**Figure 3:**
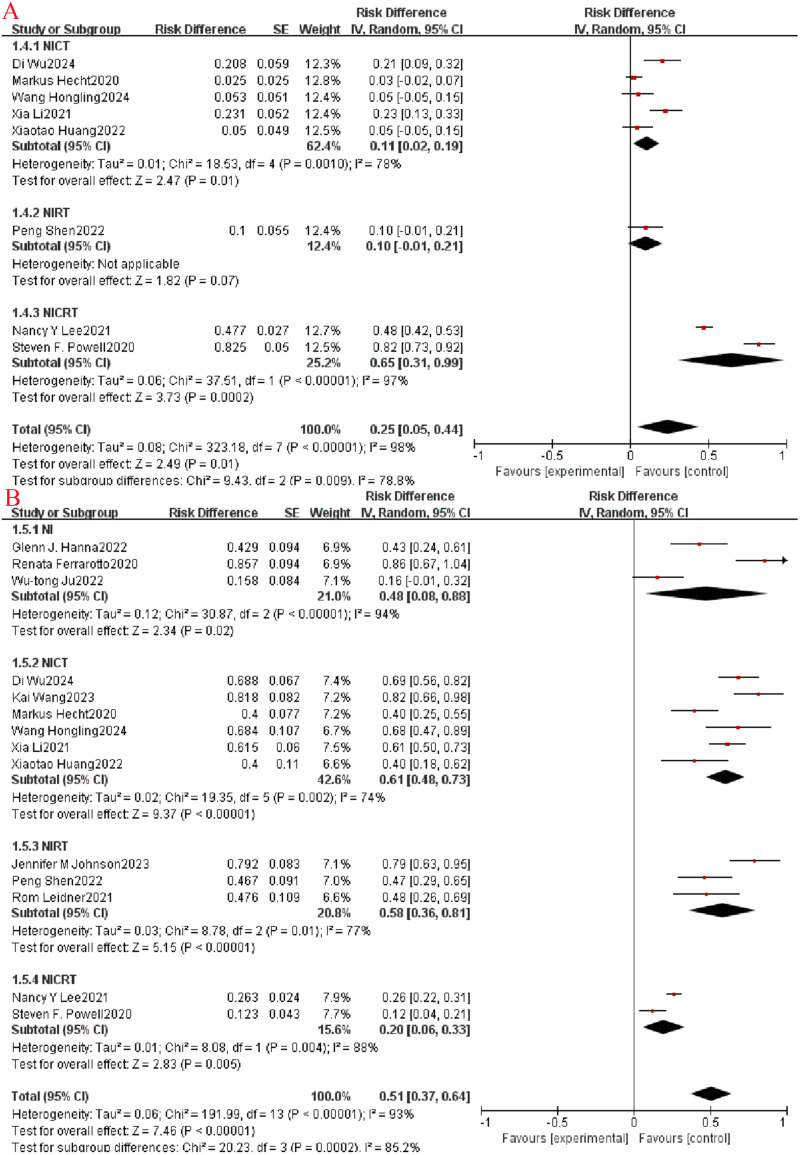
Neoadjuvant immunotherapy efficacy forest plot. **(A)**:CR;**(B)**:PR;.

**Figure 4:**
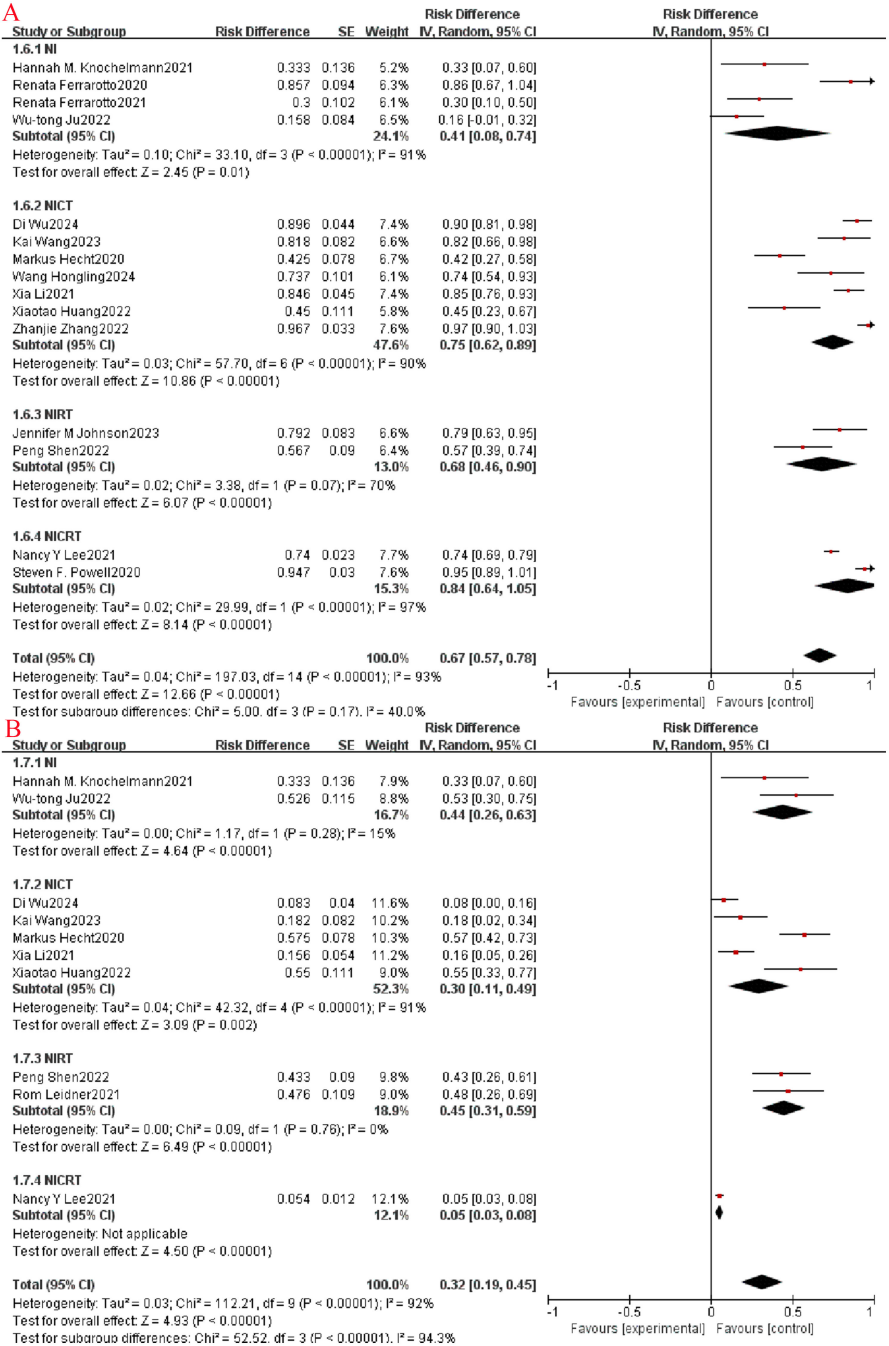
Neoadjuvant immunotherapy efficacy forest plot. **(A)**:ORR;**(B)**:SD.

Outcome metrics (CR, PR, ORR, SD, DCR) for assessing imaging in clinical trials of antineoplastic agents were performed using Response Evaluation Criteria in Solid Tumors (RECIST) version 1.1. Among the included studies, subgroup analysis revealed a higher CR rate in NICRT than in the NICT and NIRT groups (OR=0.65, 95% CI: 0.31-0.99, P= 0.009, I^2^ = 78.8%, **Figure 3A**). Meanwhile, the PR rate in the NICT group was higher than the other three groups (OR=0.61, 95% CI: 0.48-0.73, P= 0.0002, I^2^ = 85.2%, **Figure 3B**). When evaluating ORR, the NICRT group exhibited a slightly higher ORR rate (OR=0.84, 95% CI: 0.64-1.05, P=0.17, I^2^ = 40%, as shown in **Figure 4A**) compared to the other three groups, although this variance did not reach statistical significance. Regarding the SD rate assessment, the NIRT and NI groups demonstrated higher rates overall compared to the other groups (P<0.00001, I^2^ = 94.3%, **Figure 4B**). Notably, in evaluating the DCR, it was observed that three studies in the NICT group and one study in the NIRT group achieved a 100% DCR rate.

#### R0 resection rate and surgical resection rate

**Figure 5:**
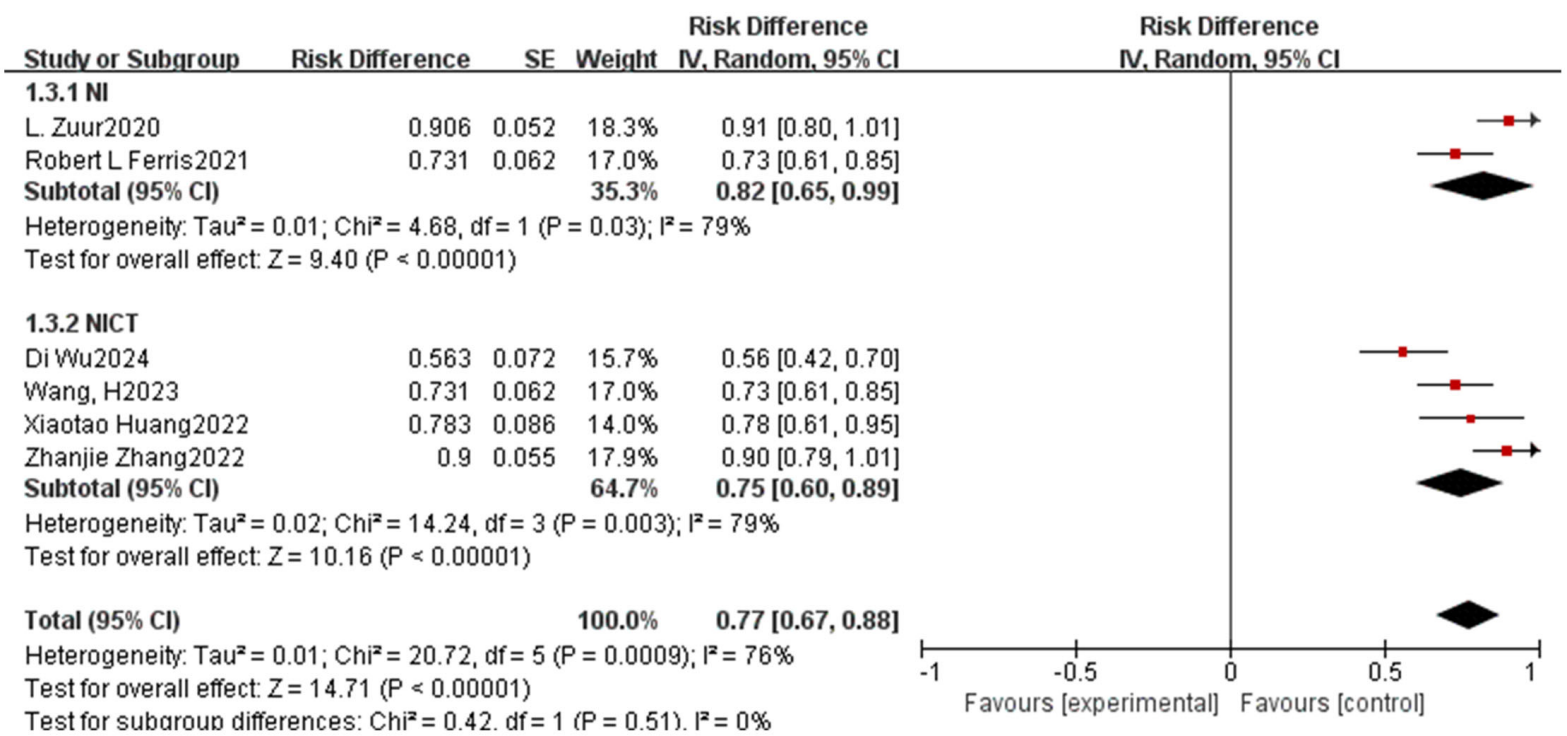
Neoadjuvant immunotherapy efficacy forest plot. **(A)** Surgical resection rate.

The R0 resection rate and surgical resection rate serve as crucial metrics for evaluating the efficacy of neoadjuvant immunotherapy. Across the included studies, the average R0 resection rate in the NI group stood at 98.9%, surpassing the rates of 93.3% in the NICT group and 90% in the NIRT group. Moreover, the surgical resection rates in the NI and NICT groups were similar, with a non-significant difference (P=0.51, I^2^ = 0%, **Figure 5**).

### Safety of neoadjuvant immunotherapy

**Figure 6:**
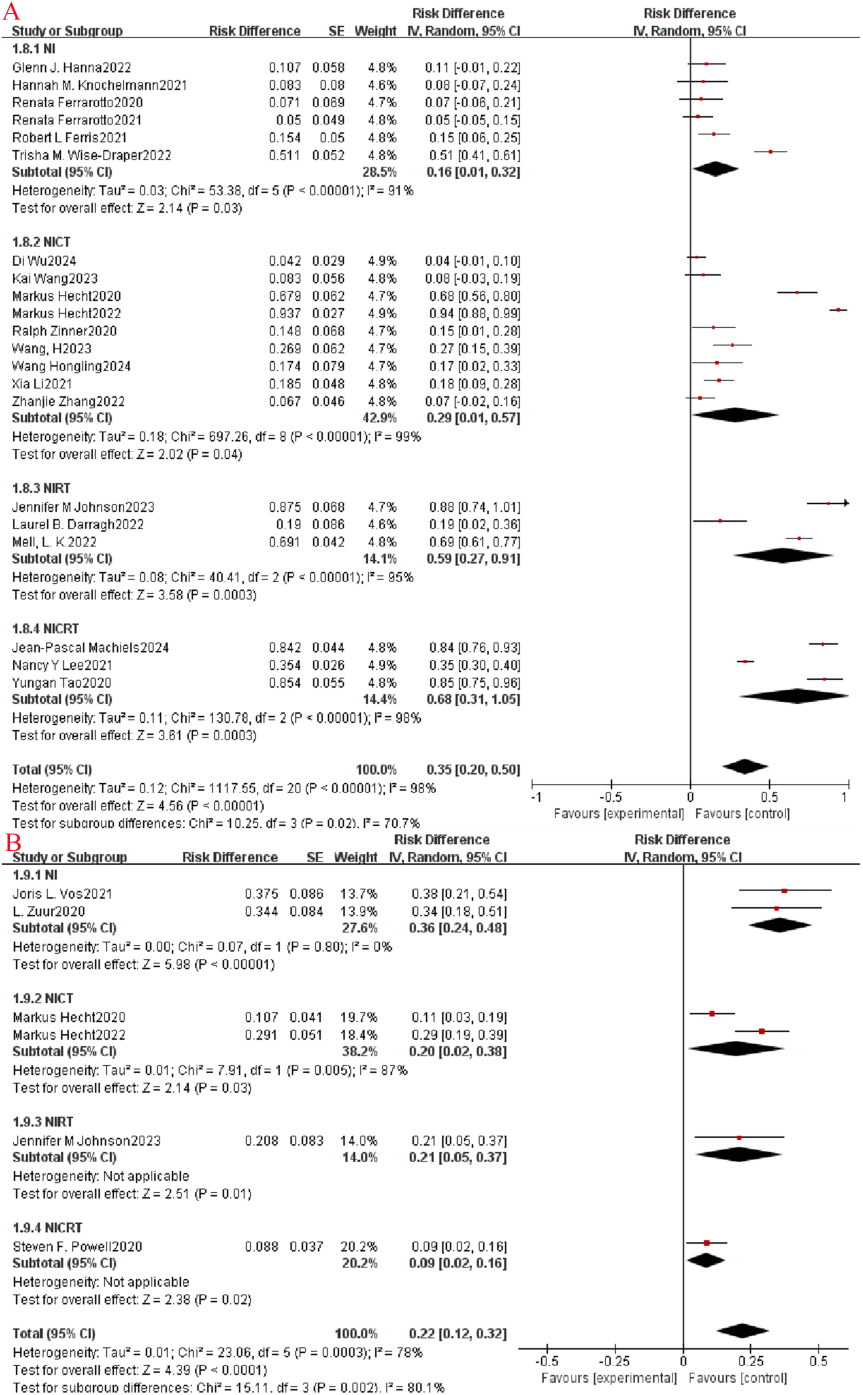
Neoadjuvant immunotherapy safety forest plot. **(A)**: ≥3 TRAEs; **(B)**: ≥3 irAEs.

The safety profile of neoadjuvant immunotherapy was evaluated based on the occurrence of grade 3-5 treatment-related adverse events (TRAEs) as outlined in the National Cancer Institute Common Terminology Criteria for Adverse Events (NCICTCAE16; version 4.0). Among the included clinical studies, 21 reported the frequency of grade 3 and higher adverse events. Subgroup analysis revealed a higher incidence of grade ≥3 TRAEs in the NICRT group compared to the other three groups (OR=0.65, 95% CI: 0.31-0.99, P=0.009, I^2^ = 78.8%, **Figure 6A**). Furthermore, 7 studies were analyzed for the occurrence of grade ≥3 immune-related adverse events (irAEs), showing that the incidence was higher in the NI group than in the other three groups (OR=0.36, 95% CI: 0.24-0.48, P=0.002, I^2^ = 80.1%, **Figure 6B**).

### Sensitivity analysis

Despite revisiting the study search, selection, and inclusion criteria, heterogeneity persisted without reduction. To ensure that the outcomes were not unduly impacted by any specific group, a sensitivity analysis was conducted by rearranging the included studies out of sequence. In the examination of individual studies on MPR, the NI group emerged as a key contributor to heterogeneity, despite not carrying the largest weight among all studies. Notably, heterogeneity significantly decreased upon excluding studies from the NI group, yet no statistically significant variance in MPR rates was observed between the NICT and NIRT groups (P=0.31, I^2^ = 1.5%, **Supplementary Figure 1**). Similarly, the NICRT group played a pivotal role in the heterogeneity of PR and SD. Following their exclusion, the PR and SD rates in the remaining three groups did not exhibit statistically significant differences (P=0.84, I^2^ = 0%, **Supplementary Figure 2**; P=0.40, I^2^ = 0%, **Supplementary Figure 3**).

Furthermore, during the sensitivity analysis investigating the safety of neoadjuvant immunotherapy, the NI group was identified as a source of heterogeneity for both the incidence of grade ≥3 TRAEs and irAEs. Upon excluding the NI group, it was revealed that the incidence of grade ≥3 TRAEs and grade ≥3 irAEs within the remaining three groups also did not show statistically significant differences (P=0.18, I^2^ = 41.2%, **Supplementary Figure 4**; P=0.28, I^2^=21.9%, **Supplementary Figure 5**).

## Discussion

Neoadjuvant therapy using immune checkpoint inhibitors has shown promise across a range of cancer types, including melanoma (41), non-small cell lung cancer (42), and bladder cancer (43). PD-1 inhibitors, specifically nivolumab and pembrolizumab, have been sanctioned for treating recurrent/metastatic HNSCC, showcasing extended OS in contrast to chemotherapy (44–46). Ongoing clinical trials have investigated neoadjuvant immunotherapies, either as standalone treatments or in combination with other medications. This meta-analysis represents the pioneering effort to assess the effectiveness and safety of various neoadjuvant immunotherapy combinations in treating patients with locally advanced HNSCC. Drawing from 32 concise studies involving 1,943 patients, our analysis quantitatively amalgamates the efficacy and safety data concerning neoadjuvant immunotherapy. Through direct subgroup analyses and sensitivity assessments, we observed that both the NICT group (OR=0.62, 95% CI: 0.41-0.84) and the NIRT group (OR=0.76, 95% CI: 0.60-0.91) surpassed the NI group (OR=0.11, 95% CI: 0.05-0.17) in achieving a higher MPR rate. However, there was no statistically significant variance between the NICT and NIRT groups. No statistically significant difference was observed in the pCR rates among the NI, NICT, and NIRT groups upon calculation. When evaluating the clinical imaging outcome metrics, we observed that the NICRT group (OR=0.65, 95% CI: 0.31-0.99) outperformed the NICT group (OR=0.11, 95% CI: 0.02-0.19) and the NIRT group (OR=0.10, 95% CI: -0.01-0.21) in terms of achieving a CR rate. However, there was no statistically significant difference in the PR rate and SD rate among the NI, NICT, and NIRT groups, although they remained higher than the NICRT group. When examining the ORR, while there were numerical discrepancies among the four groups, no statistical differences were detected. ORR serves as a valuable clinical parameter for assessing tumor treatment response through imaging; however, it has limitations, especially in the context of immunotherapy. Inflammatory pseudotumor presents histologically as a benign process characterized by acute and chronic inflammatory cells, exhibiting similar imaging features (47). This occurrence is frequently observed in patients undergoing immunotherapy, attributed to the immune impact of PD-1 inhibitors. The solid mass comprises both tumor and immune cells, resulting in a skewed assessment of ORR. Once more, there was no statistically significant variance in surgical resection rates between the NI and NICT groups.

Furthermore, this meta-analysis evaluating the safety of various neoadjuvant immunotherapies revealed that the NI group exhibited significantly lower rates of grade ≥3 TRAEs compared to the other three groups, while showing notably higher rates of grade ≥3 irAEs than the other three groups. However, no statistical differences were found between the NICT, NIRT, and NICRT groups concerning both grade ≥3 TRAEs and grade ≥3 irAEs. Treatment-related deaths, attributed to general disease, site conditions, and vascular rupture, were identified in a single study. In this study, two patients in the avelumab group experienced such events, while one patient in the placebo group passed away due to acute respiratory failure (12). Other largely controllable adverse events, including hypothyroidism, fatigue, nausea, diarrhea, oral and non-oral pain, rash/psoriasis, myalgia, constipation, cough, elevated creatinine, dyspnea, back spasms, and hypertension, as well as immune-related colitis, hyperbilirubinemia, thrombocytopenia, and proteinuria, did not lead to severe adverse consequences or increased postoperative mortality rates.The main clinical outcomes for patients with tumors are overall survival (OS) and progression-free survival (PFS), both crucial measures assessing the clinical benefits achieved by the patient. In a study by Xia Li et al., the 2-year PFS was 27% (95% CI: 18-36%) in the NI group and 44% (95% CI: 32-56%) in the NICT group, showing a statistically significant difference (P = 0.041). The 2-year OS rates in the NI and NICT groups were 61% (95% CI: 52-70%) and 70% (95% CI: 60-80%), with no statistically significant difference (P = 0.681) (27). The studies included in our meta-analysis had relatively brief follow-up durations. Consequently, the identification of superior treatment options would be facilitated by the availability of randomized controlled trials (RCTs) reporting clinical outcomes over three to five years.

Surgical resection typically stands as the primary option for locally advanced HNSCC (3). A notable ORR post-neoadjuvant therapy indicates a reduced tumor burden, making it conducive for surgical intervention. The scope of surgical resection is guided by pre-neoadjuvant imaging assessments. Further exploration is warranted to ascertain if post-treatment imaging can inform adjustments to the surgical approach and if patients achieving CR can be managed with radiotherapy alone, bypassing surgery. HPV infection serves as a significant oncogenic factor in HNSCC and is recognized as a positive prognostic indicator for the survival of HNSCC patients undergoing conventional chemotherapy and radiotherapy. Through transcriptomic analysis of 280 HNSCC cases from the TCGA database, it was observed that HPV-positive tumors demonstrated heightened immunogenicity compared to HPV-negative tumors, characterized by increased infiltration of activated CD8+ T cells (1). This underscores the role of HPV infection in stimulating the immune response. Nevertheless, the extent to which HPV-infected patients may derive greater benefits from immunotherapy remains largely unexplored.

### Limitations

The meta-analysis faced several limitations. Firstly, a portion of the data included was derived from ongoing trials or conference abstracts. Secondly, the absence of key indicators in the studies and the absence of randomized clinical trials were significant drawbacks. Moreover, the diversity in treatment protocols, use of different immunotherapeutic agents, variations in primary tumor sites, HPV status, and patient characteristics all contributed to heterogeneity, potentially diminishing the robustness of the conclusions. Furthermore, the assessment of treatment safety should encompass surgical complexity and postoperative complications. Lastly, the systematic reporting of long-term prognostic factors like OS was lacking.

## Conclusion

This systematic review and meta-analysis indicate that patients with locally advanced HNSCC might benefit from neoadjuvant immunotherapy, particularly when used in conjunction with chemotherapy or radiotherapy. Nonetheless, additional data is required to definitively confirm its efficacy.

## Data Availability

The original contributions presented in the study are included in the article/**Supplementary Material**. Further inquiries can be directed to the corresponding author.

## Supplementary material

Supplementary Figure 1 Sensitivity analysis of MPR.

Supplementary Figure 2 Sensitivity analysis of PR.

Supplementary Figure 3 Sensitivity analysis of SD.

Supplementary Figure 4 Sensitivity analysis of ≥3 TRAEs.

Supplementary Figure 5 Sensitivity analysis of ≥3 irAEs.
