# Supplementary figures and images for: Evaluating the efficacy and safety of different neoadjuvant immunotherapy combinations in locally advanced HNSCC: a systematic review and meta-analysis
Source: Front Immunol. 2024 Aug 29;15:1467306. doi: 10.3389/fimmu.2024.1467306 (PMC11390592; doi:10.3389/fimmu.2024.1467306)

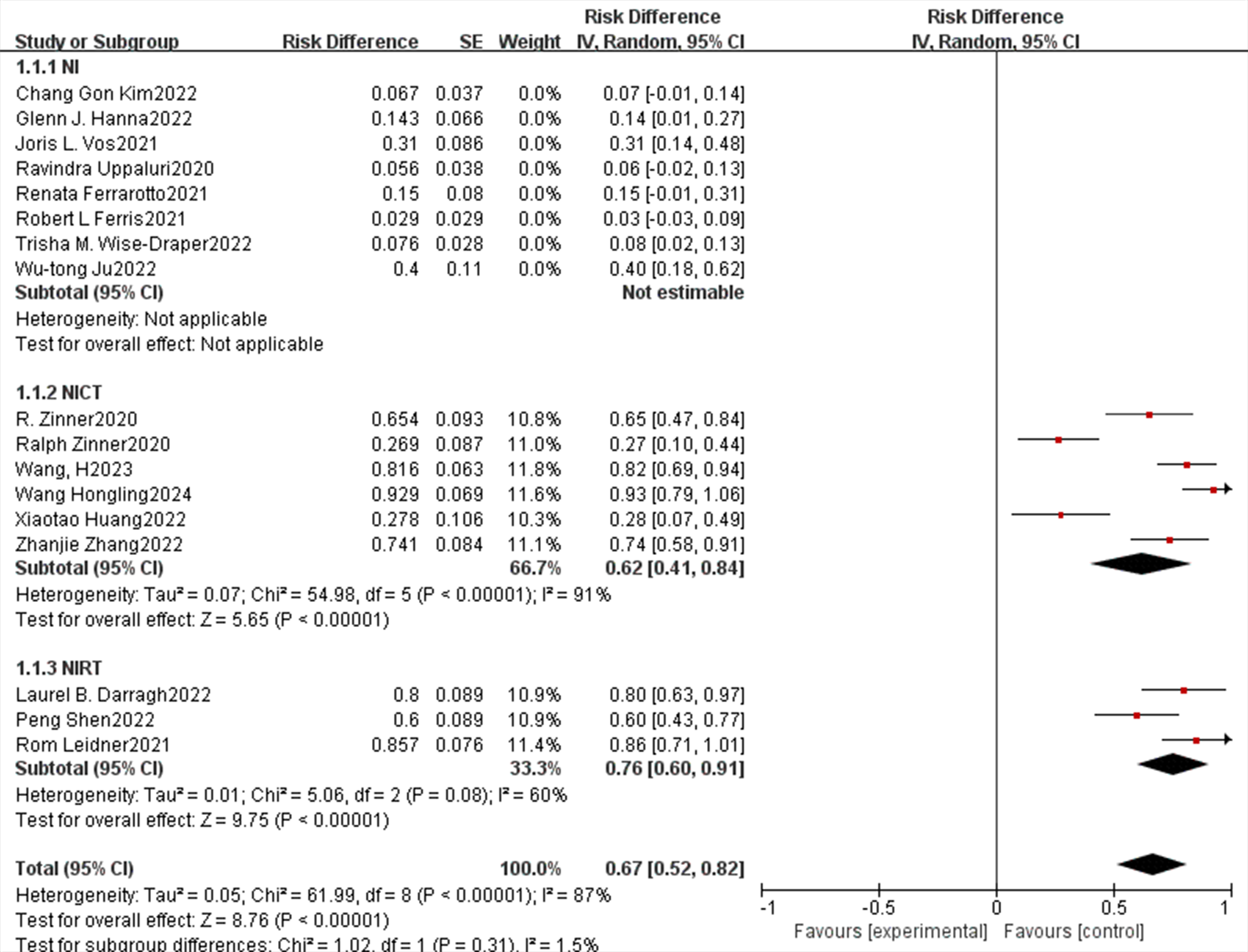

Supplement: Supplementary Figure 1 — Sensitivity analysis of MPR. [file Image1.tif]

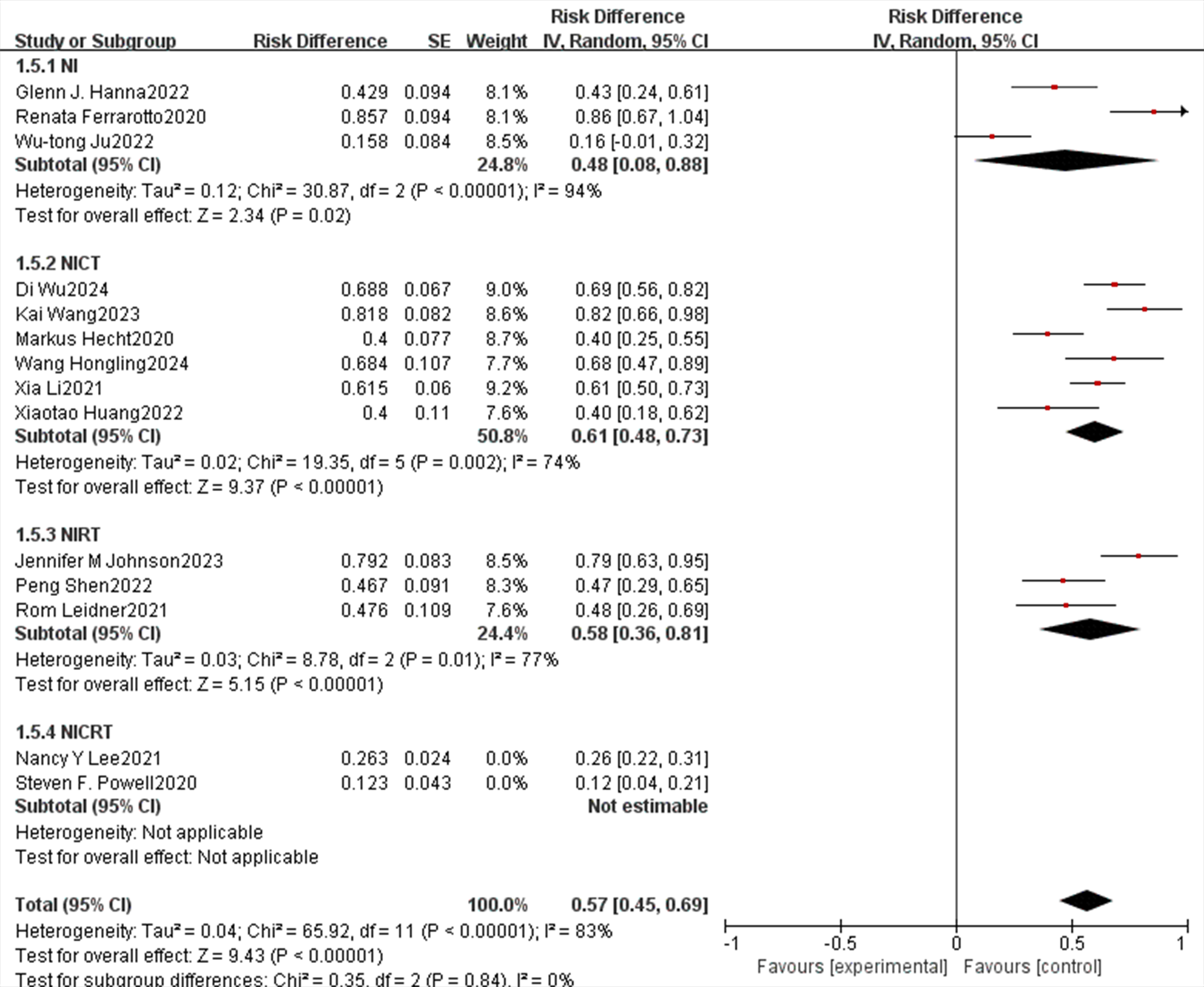

Supplement: Supplementary Figure 2 — Sensitivity analysis of PR. [file Image2.tif]

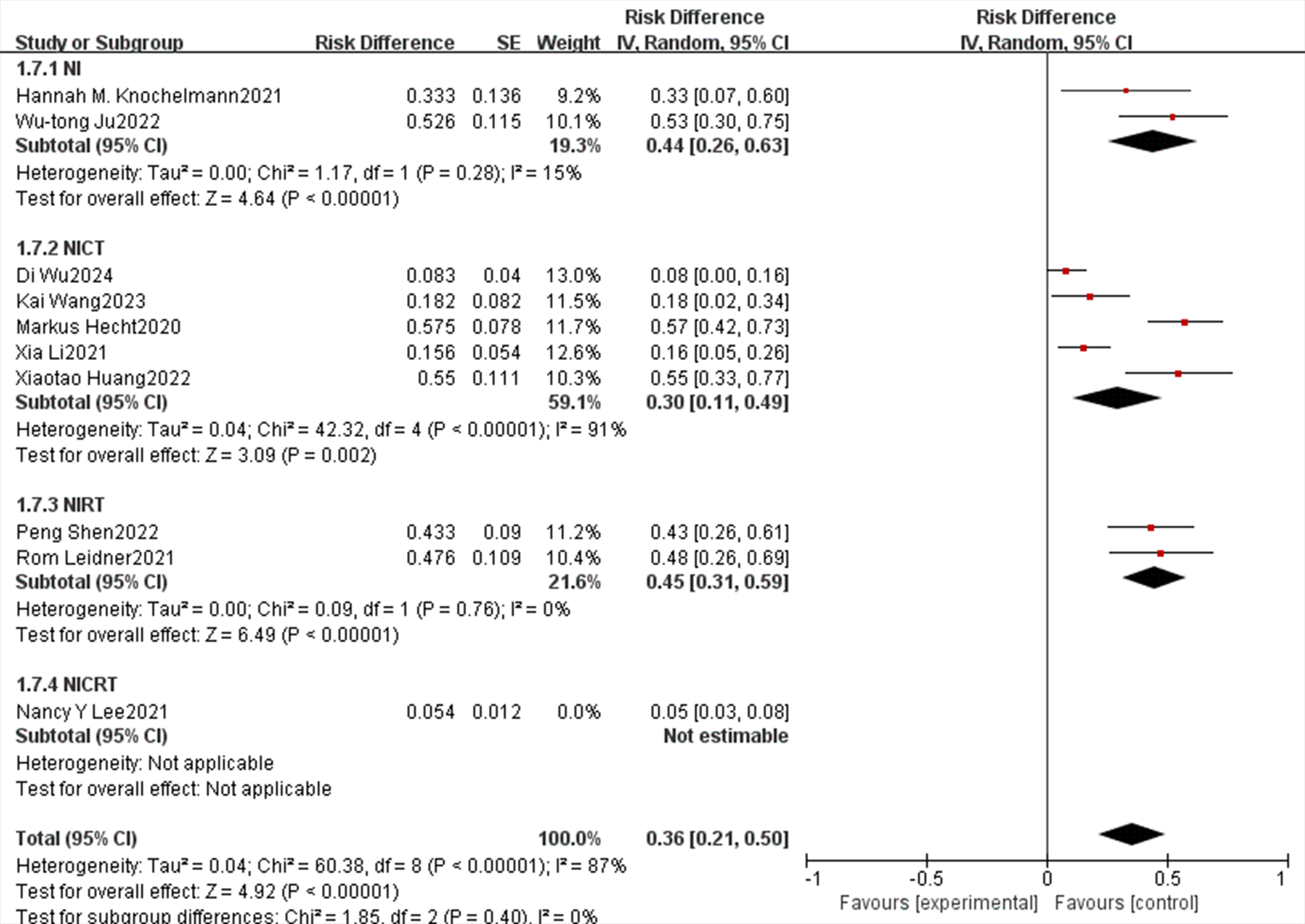

Supplement: Supplementary Figure 3 — Sensitivity analysis of SD. [file Image3.tif]

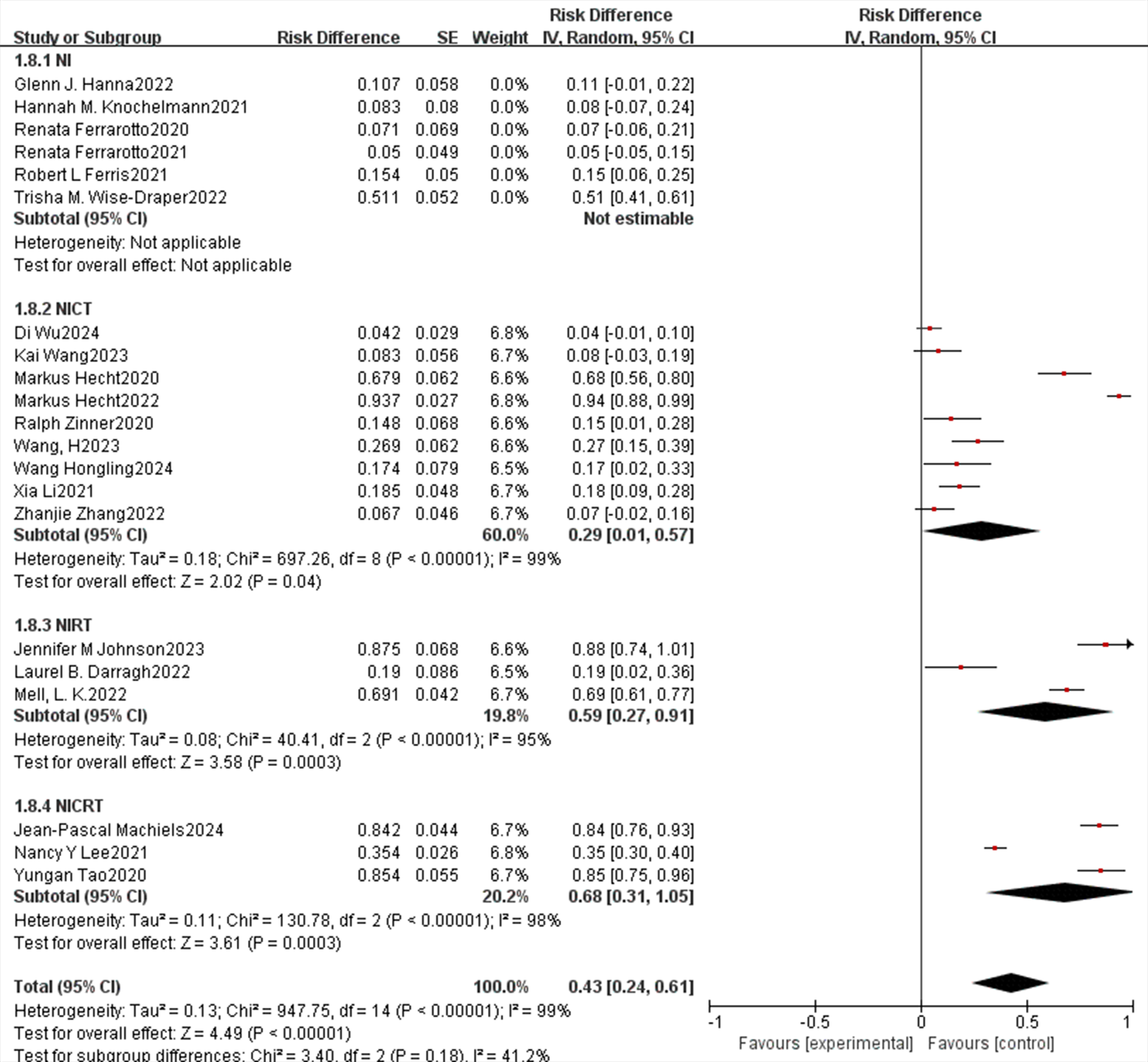

Supplement: Supplementary Figure 4 — Sensitivity analysis of ≥3 TRAEs. [file Image4.tif]

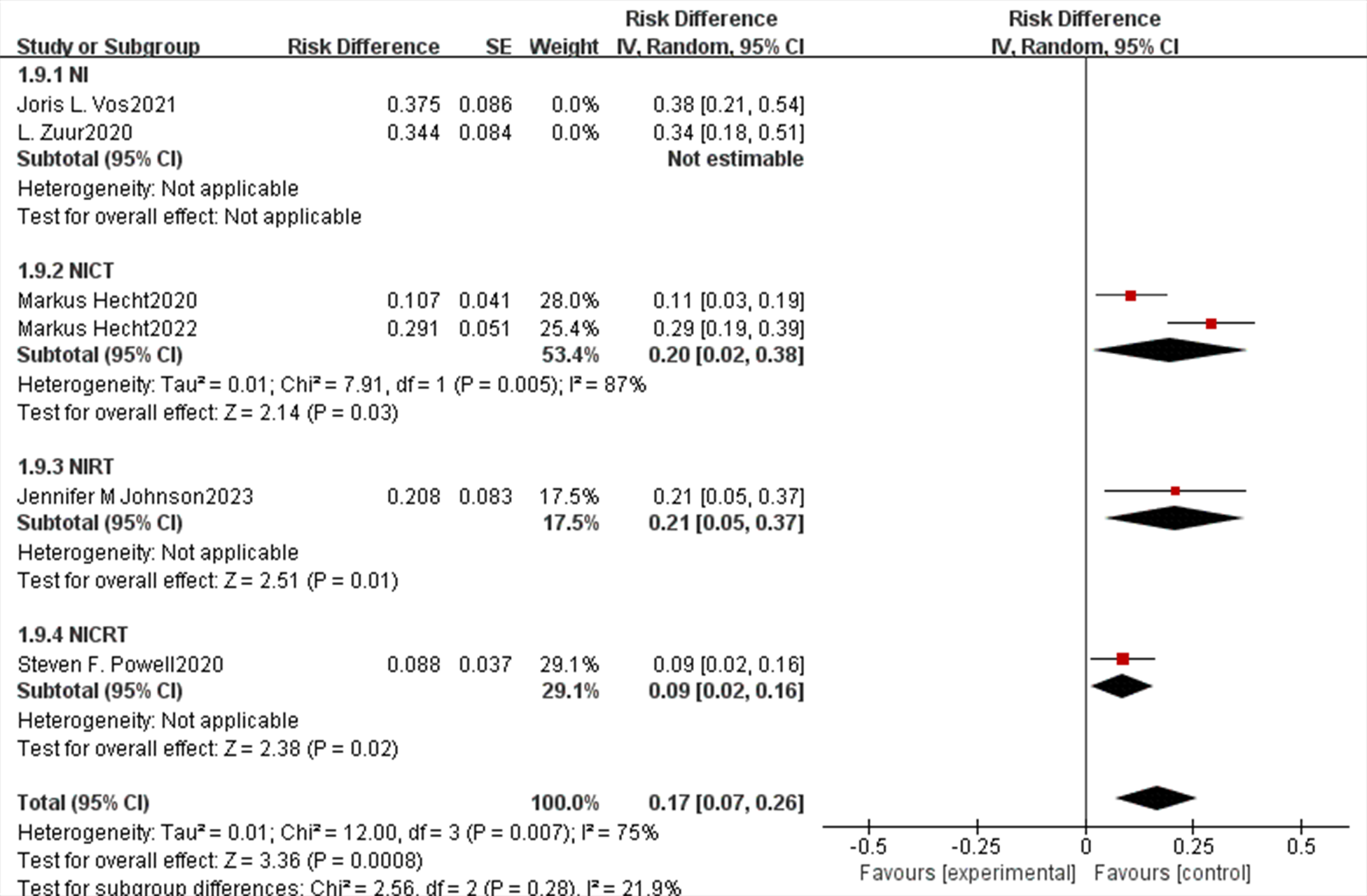

Supplement: Supplementary Figure 5 — Sensitivity analysis of ≥3 irAEs. [file Image5.tif]
