# Supplementary material for: Evaluating the efficacy and safety of different neoadjuvant immunotherapy combinations in locally advanced HNSCC: a systematic review and meta-analysis
Source: Front Immunol. 2024 Aug 29;15:1467306. doi: 10.3389/fimmu.2024.1467306 (PMC11390592; doi:10.3389/fimmu.2024.1467306)
Supplement: Supplementary file 6 [file Table1.docx]

Supplementary Table 1 Main features included in the studies

| Author  year | pCR | MPR | R0  resection rate | Surgical resection rate | CR | PR | ORR | SD | DCR | ≥3TRAEs | ≥3irAE |
| --- | --- | --- | --- | --- | --- | --- | --- | --- | --- | --- | --- |
| Renata Ferrarotto2020 |  |  |  |  |  | 12/14 | 12/14 |  |  | 1/14 |  |
| Ravindra Uppaluri2020 | 0/36 | 2/36 |  |  |  |  |  |  |  | 0/36 |  |
| L. Zuur 2020 | 9/29 |  |  | 29/32 |  |  |  |  |  |  | 11/32 |
| Renata Ferrarotto2021 | 11/20 | 3/20 | 20/20 |  |  |  | 6/20 |  |  | 1/20 |  |
| Robert L Ferris2021 |  | 1/34 |  | 38/52 |  |  |  |  |  | 8/52 |  |
| Hannah M. Knochelmann2021 | 0/12 |  | 12/12 |  |  |  | 4/12 | 4/12 |  | 1/12 |  |
| Joris L. Vos2021 |  | 9/29 | 28/29 |  |  |  |  |  |  |  | 12/32 |
| Glenn J. Hanna2022 |  | 4/28 |  |  |  | 12/28 |  |  |  | 3/28 |  |
| Wu-tong Ju2022 |  | 8/20 |  |  |  | 3/19 | 3/19 | 10/19 | 13/19 |  |  |
| Trisha M. Wise-Draper2022 |  | 7/92 |  |  |  |  |  |  |  | 47/92 |  |
| Chang Gon Kim2022 | 2/45 | 3/45 |  |  |  |  |  |  |  |  |  |
| R. Zinner2020 | 11/26 | 17/26 | 26/26 |  |  |  |  |  |  |  |  |
| Markus Hecht2020 | 27/56 |  |  |  | 1/40 | 16/40 | 17/40 | 23/40 | 40/40 | 38/56 | 6/56 |
| Konstantin Hellwig 2021 | 15/22 |  |  |  |  |  |  |  |  |  |  |
| Xia Li2021 |  |  | 20/27 |  | 15/65 | 40/65 | 55/65 | 7/45 | 62/65 | 12/65 |  |
| Markus Hecht2022 | 41/79 |  |  |  |  |  |  |  |  | 74/79 | 23/79 |
| Xiaotao Huang2022 | 3/18 | 5/18 | 18/18 | 18/23 | 1/20 | 8/20 | 9/20 | 11/20 | 20/20 |  |  |
| Zhanjie Zhang2022 | 10/27 | 20/27 | 25/27 | 27/30 |  |  | 29/30 |  |  | 2/30 |  |
| Kai Wang2023 | 8/22 |  | 22/22 |  |  | 18/22 | 18/22 | 4/22 | 22/22 | 2/24 |  |
| Di Wu2024 |  |  |  | 27/48 | 10/48 | 33/48 | 43/48 | 4/48 | 47/48 | 2/48 |  |
| Ralph Zinner2020 | 11/26 | 7/26 |  | 27/27 |  |  |  |  |  | 4/27 |  |
| Wang, H2023 | 21/38 | 31/38 |  | 38/52 |  |  |  |  |  | 14/52 |  |
| Wang Hongling2024 | 8/14 | 13/14 |  |  | 1/19 | 13/19 | 14/19 |  |  | 4/23 |  |
| Rom Leidner2021 | 14/21 | 18/21 |  | 21/21 |  | 10/21 |  | 10/21 |  |  |  |
| Laurel B. Darragh2022 |  | 16/20 |  |  |  |  |  |  |  | 4/21 |  |
| Peng Shen2022 | 10/30 | 18/30 | 27/30 |  | 3/30 | 14/30 | 17/30 | 13/30 | 30/30 |  |  |
| Jennifer M Johnson2023 |  |  |  |  |  | 19/24 | 19/24 |  |  | 21/24 | 5/24 |
| Mell, L. K.2022 |  |  |  |  |  |  |  |  |  | 85/123 |  |
| Steven F. Powell2020 |  |  |  |  | 47/57 | 7/57 | 54/57 |  |  |  | 5/57 |
| Yungan Tao2020 |  |  |  |  |  |  |  |  |  | 35/41 |  |
| Nancy Y Lee2021 |  |  |  |  | 167/350 | 92/350 | 259/350 | 19/350 | 278/350 | 124/350 |  |
| Jean-Pascal Machiels2024 |  |  |  |  |  |  |  |  |  | 335/398 |  |
