# Supplementary material for: Evaluating the efficacy and safety of different neoadjuvant immunotherapy combinations in locally advanced HNSCC: a systematic review and meta-analysis
Source: Front Immunol. 2024 Aug 29;15:1467306. doi: 10.3389/fimmu.2024.1467306 (PMC11390592; doi:10.3389/fimmu.2024.1467306)
Supplement: Supplementary file 7 [file Table2.docx]

**Supplementary Table 2** Assessment of non-randomized controlled trials in the version of MINORS.

| Study | A clearly  stated  aim | Inclusion of  consecutive  patients | Prospective  collection of  data | Endpoint  appropriate  to the study  aim | Unbiased  assessment  of endpoints | Follow-up  period  appropriate  to the major  endpoint | Loss to  follow  up not  exceeding  5% | Prospective  calculation  of the study  size | Total score |
| --- | --- | --- | --- | --- | --- | --- | --- | --- | --- |
| Renata Ferrarotto2020 | 2 | 2 | 2 | 2 | 2 | 2 | 2 | 1 | 15 |
| Ravindra Uppaluri2020 | 2 | 2 | 2 | 2 | 2 | 2 | 2 | 1 | 15 |
| L. Zuur 2020 | 2 | 2 | 2 | 2 | 2 | 0 | 2 | 0 | 12 |
| Renata Ferrarotto2021 | 2 | 2 | 2 | 2 | 2 | 2 | 2 | 2 | 16 |
| Robert L Ferris2021 | 2 | 2 | 2 | 2 | 2 | 0 | 2 | 2 | 14 |
| Hannah M. Knochelmann2021 | 2 | 2 | 2 | 2 | 2 | 2 | 2 | 1 | 15 |
| Joris L. Vos2021 | 2 | 2 | 2 | 2 | 2 | 0 | 2 | 2 | 14 |
| Glenn J. Hanna2022 | 2 | 2 | 2 | 2 | 2 | 0 | 2 | 2 | 14 |
| Wu-tong Ju2022 | 2 | 2 | 2 | 2 | 2 | 0 | 2 | 1 | 13 |
| Trisha M. Wise-Draper2022 | 2 | 2 | 2 | 2 | 2 | 0 | 2 | 2 | 14 |
| Chang Gon Kim2022 | 2 | 2 | 2 | 2 | 2 | 0 | 2 | 2 | 14 |
| R. Zinner2020 | 2 | 2 | 2 | 2 | 2 | 0 | 2 | 2 | 14 |
| Markus Hecht2020 | 2 | 2 | 2 | 2 | 2 | 0 | 2 | 0 | 12 |
| Konstantin Hellwig 2021 | 2 | 2 | 2 | 2 | 2 | 0 | 2 | 1 | 13 |
| Xia Li2021 | 2 | 2 | 0 | 2 | 2 | 2 | 2 | 2 | 14 |
| Markus Hecht2022 | 2 | 2 | 2 | 2 | 2 | 0 | 2 | 1 | 13 |
| Xiaotao Huang2022 | 2 | 2 | 2 | 2 | 2 | 0 | 2 | 1 | 13 |
| Zhanjie Zhang2022 | 2 | 2 | 2 | 2 | 2 | 0 | 2 | 1 | 13 |
| Kai Wang2023 | 2 | 2 | 2 | 2 | 2 | 0 | 2 | 2 | 14 |
| Di Wu2024 | 2 | 2 | 2 | 2 | 2 | 0 | 2 | 2 | 14 |
| Ralph Zinner2020 | 2 | 2 | 2 | 2 | 2 | 0 | 2 | 1 | 13 |
| Wang, H2023 | 2 | 2 | 2 | 2 | 2 | 0 | 2 | 2 | 14 |
| Wang Hongling2024 | 2 | 2 | 2 | 2 | 2 | 0 | 2 | 2 | 14 |
| Rom Leidner2021 | 2 | 2 | 2 | 2 | 2 | 0 | 2 | 1 | 13 |
| Laurel B. Darragh2022 | 2 | 2 | 2 | 2 | 2 | 0 | 2 | 1 | 13 |
| Peng Shen2022 | 2 | 2 | 0 | 2 | 2 | 2 | 2 | 2 | 14 |
| Jennifer M Johnson2023 | 2 | 2 | 2 | 2 | 2 | 2 | 2 | 1 | 15 |
| Mell, L. K.2022 | 2 | 2 | 2 | 2 | 2 | 0 | 2 | 1 | 13 |
| Steven F. Powell2020 | 2 | 2 | 2 | 2 | 2 | 0 | 2 | 1 | 13 |
| Yungan Tao2020 | 2 | 2 | 2 | 2 | 2 | 0 | 2 | 2 | 14 |
| Nancy Y Lee2021 | 2 | 2 | 2 | 2 | 2 | 2 | 2 | 2 | 16 |
| Jean-Pascal Machiels2024 | 2 | 2 | 2 | 2 | 2 | 0 | 2 | 1 | 13 |
